# Sustainable Perforated Acoustic Wooden Panels Designed Using Third-Degree-of-Freedom Bezier Curves with Broadband Sound Absorption Coefficients

**DOI:** 10.3390/ma16186089

**Published:** 2023-09-06

**Authors:** Bartlomiej Chojnacki, Kamil Schynol, Mateusz Halek, Alicja Muniak

**Affiliations:** 1 Department of Mechanics and Vibroacoustics, AGH University of Science and Technology, Mickiewicza Av. 30, 30-059 Cracow, Poland; 2 Form At Wood sp. z o.o., Technologiczna 2, 45-839 Opole, Poland; k.schynol@formatwood.com (K.S.); m.halek@formatwood.com (M.H.); 3 Soundway Acoustics, Owocowa 17, 26-600 Radom, Poland

**Keywords:** sound absorbers, impedance tube measurement, reverberation chamber, perforated panel modeling, FEM acoustic modeling

## Abstract

The current interior design scope places high demands on acoustic treatment manufacturers. The state of the art does not provide satisfactory material proposals for architects to satisfy design needs. There is a need for a novel approach concerning decorative, recognized materials that adapts them to the acoustic surface properties. The final design proposed in this study presents a modern functional solution with high acoustic properties, which can be produced with sustainable materials such as FSC wood and has a low environmental impact because of its low waste production. This research presents the complete design process of a novel type of wooden acoustic panel. A comprehensive explanation of the scientific development is covered, including basic material testing in an impedance tube, FEM simulations of the initial designs, and final measurements in a reverberation chamber. The solution’s novelty is based on the optimized placement of the perforation holes on the surface of a wooden overlay using a ship deck optimization algorithm. The methods used cover the original solution of mixing FEM modeling of the surface impedance with the application of the Jeong–Thomasson correction for random incidence sound absorption coefficient simulation. The contribution of this research is the development of wooden perforated panels with Class A sound absorption and an overall depth of 90 mm, including the 50 mm depth of the backing material. The discussion will explain the difficulties of working with this material and the need for a combination of the aesthetic and acoustic sides of the project.

## 1. Introduction, Research Background, and Conception

### 1.1. Introduction

One widely used acoustic material for sound absorption is perforated panels based on the Helmholtz resonator concept. Each hole in a panel acts as a separate Helmholtz resonator, where the hole is a neck and the panel distance from the rigid wall is the volume of the resonator [1]. Air voids are often filled with sound-absorbing materials—mainly mineral or glass wool—broadening the effective absorption range. However, perforated panels’ popularity is based on the economics of their use and their construction functions. Typical perforated panels with regular perforation patterns do not meet the current aesthetic needs in the modern interior design market, and novel material development is needed. Acoustic engineers frequently use acoustic perforated panels because of their flexibility in shaping their absorption characteristics with hole size and diameter modifications. However, irregular hole placement is not common [2,3]. The basic concepts of novel types of perforated panels will be explained with a comprehensive description of the following features:

- The scientific method used to reach the desired broadband sound absorption;
- The influence of the third-degree-of-freedom front face of the wooden panel;
- The possibilities of final panel property manipulation with the exchange of the front wooden overlays.

The sound absorption potential of a panel based on third-degree curves was investigated in this paper. Based on the Form At Wood wooden overlays with scientific design and modeling from the AGH University of Science and Technology in Cracow, a novel type of perforated acoustic panel with high broadband absorption was developed.

This article provides a comprehensive overview of the step-by-step process of creating acoustic panels utilizing third-degree overlays. Section 1 delves into the conceptualization stage, where research on this topic was conducted to obtain an overview of the available solutions and background on the technical aspects of this structure. Section 2 describes the results of samples measured in an impedance tube, delivering information about the sound absorption coefficients of flat and 3D panels. This step provided valuable insights for further improvement and investigation. Section 3 explores the possibilities of perforated panels using the finite element method (FEM) in COMSOL Multiphysics 5.3, where various scenarios were tested. This stage allowed the researchers to fine-tune the design parameters and verify the perforation rate. Section 4 discusses the reverberation chamber measurements of the final products. These measurements served as a crucial validation step, ensuring that the actual acoustic performance of the manufactured panels aligns with the expected specifications. Section 5 summarizes the development phase findings and the possible influence of the new design on the state of the art.

### 1.2. Research Background and Available Solutions

Acoustic perforated panels are widespread in public spaces because of their acoustic and construction performances. However, most currently used designs are based on a regular hole placement in the grid [2]. They are easy to design thanks to the well-known analytical equations for perforated panel construction [4,5]. Advanced shapes in perforated panel materials are uncommon [6] as their performance is difficult to predict, while the high prototyping costs make the final reverberation chamber product testing unprofitable. Most of the current papers on acoustic panel development only cover the analytical problem formulation [7,8] or the final design measurement in the engineering reports [9,10,11]. 

The authors of [12] investigated some non-uniform perforation patterns in impedance tubes. However, no further reverberation chamber measurements were presented. Also, their research was focused only on MPPs (micro-perforated panels) with a flat shape. Reference [13] also used the non-uniform perforation pattern modeling approach to investigate the possibility of the artistic application of acoustic perforated panels. In that study, only impedance tube sound absorption measurements were presented. Very comprehensive research is found in [14], where methods similar to those adopted in this paper were used, i.e., FEM modeling and reverberation chamber measurements, confirming the possibility of the joint use of these methods. The research in [14] also focused on MPPs, but it was found that including this type of perforation in solid wood materials is complicated [15,16]. The crucial concern in similar research is how to apply radiation impedance from a non-flat surface. In this paper, we assumed the panel surface to be flat and followed a similar approach for a different material: a wooden 3D perforated panel with a porous backing. Recently, the research on sound-absorbing materials has mainly been focused on metamaterial panel development [17,18], and the methods, such as a combination of FEM modeling and impedance tube testing, can be adapted to other types of acoustic panels.

There is a lack of comprehensive process formulation in acoustic material development for wooden perforated panels with irregular perforation patterns, from the initial design to the modeling phase, including FEM modeling and the final prototype testing. Wooden 3D panels using third-degree curves are not included in the current state of the art, meaning that initial testing is required, including impedance tube material measurements, to verify the possible influence of the complex front panel shape on the acoustic absorption.

### 1.3. Research Conception of Acoustic Panels Using Third-Degree Curves

**Figure 1:**
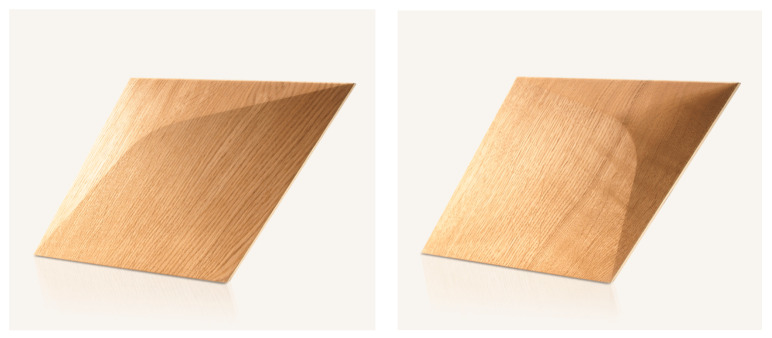
An exemplary panel created using 3rd-degree curves [20].

Third-degree curves, also known as cubic functions or cubic curves, are mathematical functions that describe curves or surfaces in three dimensions. They are represented by third-degree equations that consist of terms involving the variables x and y. The distinctive characteristics of third-degree curves are their smoothness and flexibility, enabling them to model a wide range of shapes and forms, such as arcs, circles, ellipses, and even more complex curves [19]. These curves offer high precision and aesthetic representation, making them widely utilized in various fields. In the context of this article, the discussion of third-degree curves is significant as they can be employed to represent and manipulate the design of acoustic panels by leveraging the smoothness and versatility of the curves. An exemplary panel created using third-degree curves is shown in Figure 1.

The novel shape of the proposed panels complies with modern interior design requirements and builds upon the difficulties in applying acoustic properties to these types of structures to maintain the visual and acoustic features of the product. The product was selected as a base for further acoustic panel development due to the following sustainable material properties:

- Based on wood with an FSC (Forest Stewardship Council) certificate;
- Low waste production;
- Ecological and non-toxic glues or wax oils.

The selected material types do not allow for acoustic panel formulation using micro-perforated panels or metamaterial absorbers, so a new adaptation of classic perforated panels should be investigated.

## 2. Impedance Tube Tests

### 2.1. Impedance Tube Method

The essential elements of acoustic panels, including perforated and slotted panels, are the core of the sound-absorbing material and the front panel, which can be produced using various perforations/slots. The proper configuration of these two elements is crucial for the final ability of a panel to absorb sound in a room. Previously, research was performed to assess basic wood sound absorption properties [15,16,21]. However, it did not cover the possibility of 3D front panel shapes or different wave finishing options used in practice. To measure the acoustic absorption of panels, the two-microphone impedance tube method was applied according to the ISO 10534-2 standard [22]. The hardware used was a B&K Impedance tube (type 4206), which complied with the standard requirements.

### 2.2. Basic Research of Phenomena in Perforated Panels

**Figure 2:**
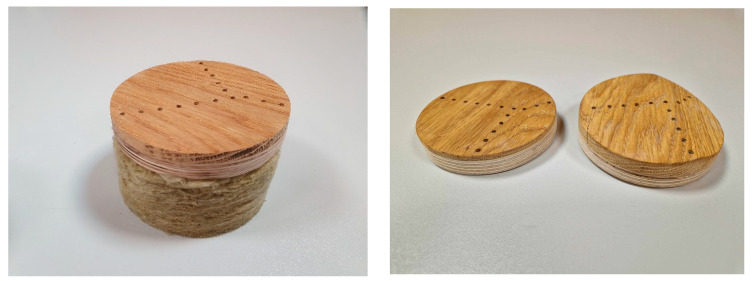
Test samples used in the impedance tube measurements.

This research’s main goal involved determining the impacts of variable perforation thicknesses and different wood finishing methods on the sound absorption coefficients of panels produced using third-degree curves. The designed overlays featured a thickness jump of up to 14 mm in the 3D model and were prepared by cutting samples with 100 mm diameters from the complete Caro Minus panel shown in Figure 1, which fit the impedance tube dimensions. All samples were tested using 50 mm recycled-material-based fiberglass as a backing material (density: 50 kg/m^3^; flow resistivity: around 32 kPa*s/m^2^). Photographs of the test samples are shown in Figure 2.

To assess the influence of the 3D front surface on the acoustic parameters of the panels, investigations were carried out in the impedance tube. Two identical variants of perforations—flat and cut from an element produced using third-degree curves (3D)—were measured together with two typical surface finishings. The following variants of the materials were used:

- Variant 1—flat panel with front surface polished;
- Variant 2—flat panel with front surface brushed;
- Variant 3—3D panel with front surface polished;
- Variant 4—3D panel with front surface brushed.

**Figure 3:**
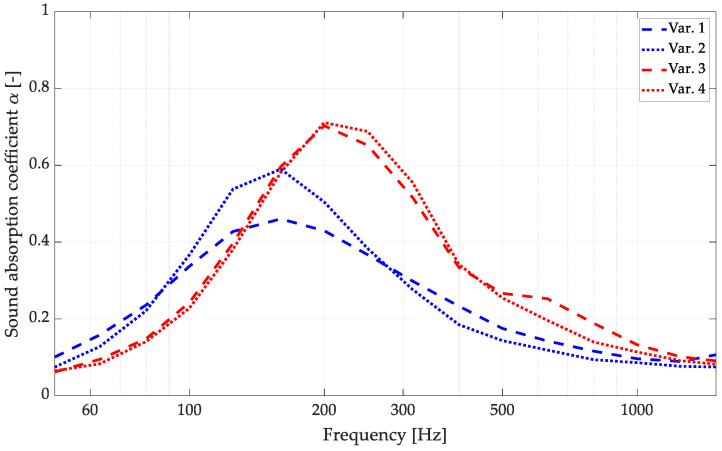
Impedance tube sound absorption measurements for different front surface finishings and the influence of the 3D panel surface.

The results of the sound absorption measurements are presented in Figure 3.

The obtained results led to the conclusion that the panels produced using third-degree curves with applied perforations exhibited slightly better acoustic properties than the flat overlays across the measured frequency range. The use of a 3D surface provided worse performance in the low-frequency range. However, the average sound absorption was improved significantly in the mid- and high-frequency ranges, which may be crucial for perforated panels where sound absorption in the highest octaves is challenging to achieve [4]. The influence of different finishing options for flat panels is significant, while for 3D panels, it seems to be neglectable. This is essential information as it would be challenging to provide a proper visual finish with the brushed surface type; therefore, the use of the polished surface type is desirable. The impedance tube tests improved the panel properties using third-degree curves and allowed us to proceed with further panel development. 

## 3. Numerical Tests of 3D acoustic Wooden Panels

### 3.1. FEM Simulations of the Primary Panel Concept and the Perforation Optimization Process

The effectiveness of perforated panels is primarily defined by parameters such as the number and size of the holes (known as the perforation rate), the plate thickness, and the amount of absorbing material behind the panel. As the plate thickness and absorbing material depth (50 mm) were defined using the preliminary assumptions for panel design, significant attention was focused on numerical simulations of the designed overlays to optimize these parameters and achieve the highest possible sound absorption coefficient across a wide frequency range. Therefore, in this stage, the focus was mainly on optimizing the number and size of the holes in the smallest possible area to preserve the aesthetics of the panels. Considering the base overlay shape (triangle and rhombus), the hexagonal shape, which merges three or six overlays, was selected for further development.

The goal of this stage was to perform the simulation closest to the final panel performance, and thus random incidence sound absorption coefficient simulations were required with the given 3D front panel pattern. The essential perforated panel formulas are based only on regular hole patterns. The typical formula for acoustic surface impedance calculation is (1) [4]:

(1) $$z_{n}=r_{m}+i\omega m+z_{n-1}$$

where *z_n_*_−1_ is the surface impedance of the previous layer (typically porous, calculated in an example from the methods described in [4]), ω is the angular frequency, and *r_m_* is the flow resistivity in the holes (2):

(2) $$r_{m}=\frac{\rho_{0}}{\varepsilon }\sqrt{8\nu \omega }\left(1+\frac{t}{2a}\right)$$

where $\rho_{0}$ is the air density (typically 1.21 kg/m^3^), ε is the perforation rate, ν is the kinematic viscosity (typically 1.84 × 10^−5^), *a* is the radius of the holes, and *t* is the base plate depth. Finally, the equation for *m*—the mass of the air inside the holes—is written as follows (3):

(3) $$m=\frac{\rho_{0}}{\varepsilon }\left(t+2\delta a+\sqrt{\frac{8\nu }{\omega }\left(1+\frac{t}{2a}\right)}\right)$$

where δ is the aperture correction dependent on the final hole shape [23,24,25]. These equations can be used for the regular type of perforation on plain plates, while they are impossible to use for 3D perforated panels with irregular shapes. Following these equations, the reflection coefficient can be calculated (4):

(4) $$R=\frac{\frac{z_{n}}{\rho_{0}c_{0}}\cos\left(\psi \right)-1}{\frac{z_{n}}{\rho_{0}c_{0}}\cos\left(\psi \right)+1}$$

along with the final sound absorption coefficient for the given wave incidence angle (ψ) (5):

(5) $$\alpha =1-{\left|R\right|}^{2}$$

**Figure 4:**
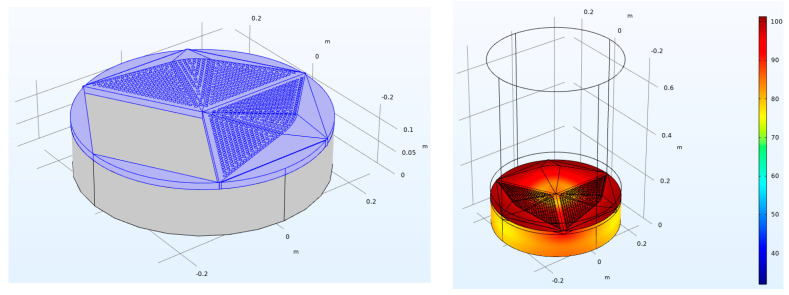
A representation of the 3D COMSOL Multiphysics 5.3 model used for advanced simulations for 3rd-degree-of-freedom acoustic wooden panels.

The goal of this research stage was to complete an assessment in the low-frequency range as it was considered crucial for further panel development. As the normal incidence or oblique angle sound absorption coefficient values are typically smaller than the final random incidence sound absorption coefficient, advanced modeling was required to simulate this case. The Jeong–Thomasson correction method was used, where the fundamental equation is defined as (6) [26,27]:

(6) $$\alpha_{size}=2\int_{0}^{\frac{\pi }{2}}\frac{4ℜ\left(Z_{S}\right)}{{\left|Z_{S}+Z_{r}\left(\theta \right)\right|}^{2}}\;sin\left(\theta \right)d\theta$$

where *Z_S_* is the normalized surface impedance, *Z_r_* is the radiation impedance calculated following the reference paper [27], and *θ* is the considered angle for integration purposes (typically in the range of 0 to *π*/2). Equation (6) is valid with the assumption of a local reaction, which applies to the current absorber as it has acoustic perforations in its base. In the current research, the FEM modeling software COMSOL Multiphysics was used to obtain the values of surface impedance (*Z_S_*) for the designed panels as it was necessary to calculate the random incidence sound absorption coefficient. The impedance tube simulation model was prepared using previously described methods [28,29]. Similar research was also presented in [17,18]. Concerning the total sample size diameter (500 mm), the upper frequency available to simulate was 550 Hz. The interface of the COMSOL model is shown in Figure 4. The model was constructed with the following modules of COMSOL to simulate all required physics following the practice described in [30,31]:

- The porous layer application of the backing material of the panel was simulated using the Delany–Bazley porous material model [4] with the flow resistivity equal to 30 kPa*s/m^2^.
- Viscous loss inside the holes was simulated with the thermoviscous acoustics module, applying the physics following [32]. Inside acoustic ports, the temperature change and viscoelastic port boundary conditions were applied, as these phenomena are essential for the overall behavior of a perforated plate. Detailed information on how to simulate the viscous loss is included in references [33,34].
- Incident sound waves were modeled using the background pressure field feature in COMSOL in the range valid for plane waves in modeled geometry. Only the normal incidence was considered for calculating the random incidence sound absorption later with the Jeong–Thomasson correction.

To calculate the surface impedance on the 3D panel, we followed previous works [35,36] and used Equation (7):

(7) $$Z_{S}=\langle \frac{p_{s}}{u_{n}}\rangle =\langle \frac{p_{s}}{u*n}\rangle$$

where *p_s_* is the surface pressure of the panel, *u_n_* is the normal velocity at the same point, ***u*** is the velocity vector at the given analysis point, *n* is the vector normal to the surface of the acoustic panel, and the symbol < > stands for the averaging operation. Following [35,36], the impedance was averaged across the panel’s whole surface, including the effects of both the perforated and non-perforated parts. For $\alpha_{size}$, a rectangular sample was used with a size of 3 × 3.5 m as it reflected the required conditions for the later ISO 354 [37] measurements.

The computational environment in [18] offered a similar solution but with an overall sample diameter of up to 100 mm, which allowed the upper simulation frequency to be 1400 Hz. In the current case, the panel sample could not be reduced as it had to reflect the possible final design of the panels, and the modeling of just a part of the panel did not provide any useful information for this research. The goal was to justify the performance with just 50 mm of backing material and an overall panel depth of 90 mm to investigate whether it is possible to use this configuration to achieve satisfactory performance in the low-frequency range, considered the most difficult in the current design.

### 3.2. Optimized Design of the Wooden Overlays

The assumption for the panel design was to maintain the visual aspects of the panel while adding sound-absorbing properties to it. To achieve this goal, it was decided to reflect the basic overlay shapes such as edges, keep them as trackers, and place as many holes as possible for the given minimum distances and panel shape. The boundary assumptions, such as panel edges, minimum distances between the holes, and hole diameters, were used in the simplified version of the ship deck arrangement algorithm, which was found to be suitable for this use [38]. The optimization parameters considered the minimum distance from the overlay edges to be 5 mm and the minimum distance between the hole edges to be 3 mm, which was assumed to be safe for panel consistency. In the initial phase of this research, three different panels were planned: two with the highest possible broadband sound absorption produced with the Pyramid and Caro Minus overlay types and one representing a lower absorption class, a resonant panel, based on the Caro Minus overlay. Multiple simulations with different patterns were performed in the FEM environment, which led to the final designs of the panels. However, the authors decided not to present them in the final paper because of the large amount of data and the significant number of resulting plots. Ultimately, it was decided to produce overlays characterized by the parameters shown in Table 1.

**Figure 5:**
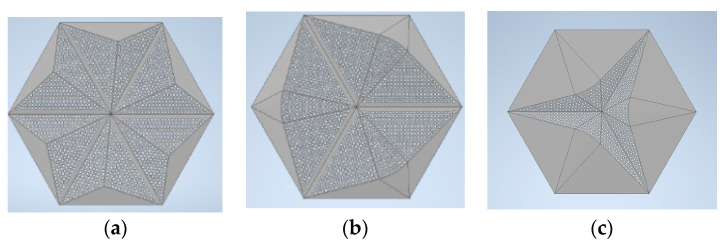
Three-dimensional models of panels showing the best efficiency: (**a**) P-A; (**b**) CM-A; (**c**) CM-D.

**Figure 6:**
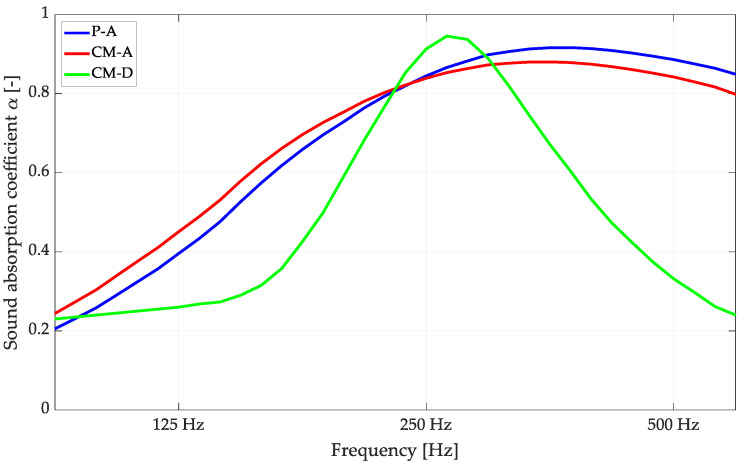
FEM calculations of sound absorption coefficients after applying Thomasson’s modification for the selected variants of 3rd-degree-of-freedom perforated overlays.

Top views of selected geometries of the 3D model are shown in Figure 5, and their calculated sound absorption coefficients after the Jeong–Thomasson correction are shown in Figure 6.

This stage of the research led to the following conclusions:

(a) A perforation rate of over 25% and a hole diameter of no less than 6 mm should be attained to achieve practical sound absorption values in the mid- and high-frequency ranges. Panels with these characteristics have the potential to obtain Class A certification.
(b) Panels with lower perforation rates can operate in a resonant mode using small holes of approximately 3 mm. However, achieving a wideband characteristic and obtaining a sound absorption class higher than E or D are impossible.

### 3.3. Variable Properties with Different Panel Arrangements

**Figure 7:**
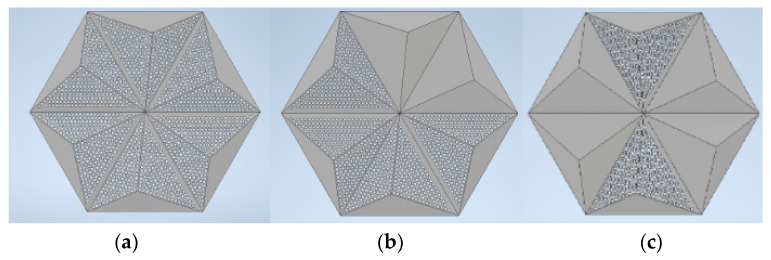
Three-dimensional models of panels in a partial P-A overlay arrangement: (**a**) 3/3 of the overlay is perforated; (**b**) 2/3 of the overlay is perforated; (**c**) 1/3 of the overlay is perforated.

**Figure 8:**
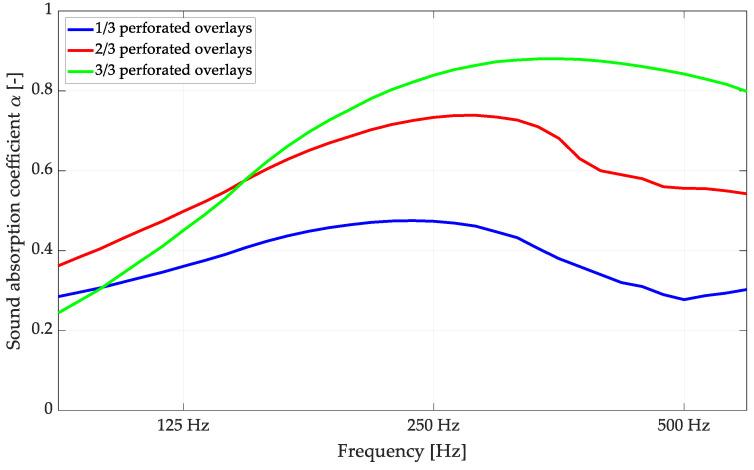
FEM calculations of sound absorption coefficients after applying Thomasson’s modification for the variable acoustic version of the P-A overlay.

Variable acoustic features are a desirable property for common materials as they allow the modification of the acoustic conditions in a room [2,39]. In order to investigate the influence of variable configurations of overlays within a single panel on the sound absorption coefficient, several analyses were conducted. As selected in this paper, the most promising was the situation when 1/3 or 2/3 of the perforated overlays closed in a hexagonal shape were replaced by standard, non-perforated panels (Figure 1). Sketches of this situation are shown in Figure 7. The effect of FEM modeling on these configurations is shown in Figure 8.

Including non-perforated overlays in a hexagonal configuration with one or two perforated overlays in the P-A pattern significantly reduced the sound absorption coefficient values. This modification can be applied easily, even after mounting in in situ conditions, which may provide unique features for variable acoustic applications with wooden perforated panels.

## 4. Reverberation Chamber Tests

### 4.1. Final 3D Perforated Panel Construction

**Figure 9:**
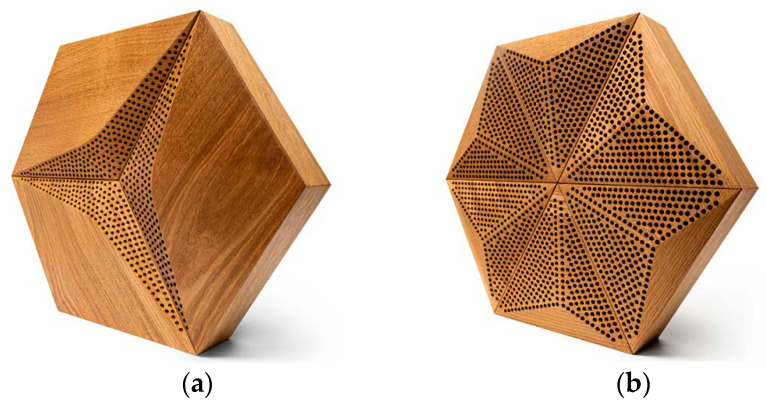
The final representations of the perforated acoustic wooden panels produced using 3rd-degree-of-freedom curves: (**a**) CM-D type; (**b**) P-A type.

The last stage of product development is certification and property validation. The most common method for testing acoustic materials is reverberation chamber sound absorption measurement according to the ISO 354 standard [37]. For this purpose, the final version of the wooden panels was prepared in a closed, hexagonal enclosure, as shown in Figure 9. The front panel wooden overlays were manufactured using a five-axis CNC milling machine, and the perforation patterns were prepared with a three-axis fast drilling machine. To maintain the practical application form, the panels were closed with backing mineral wool inside a plywood case with a 20 mm depth.

**Figure 10:**
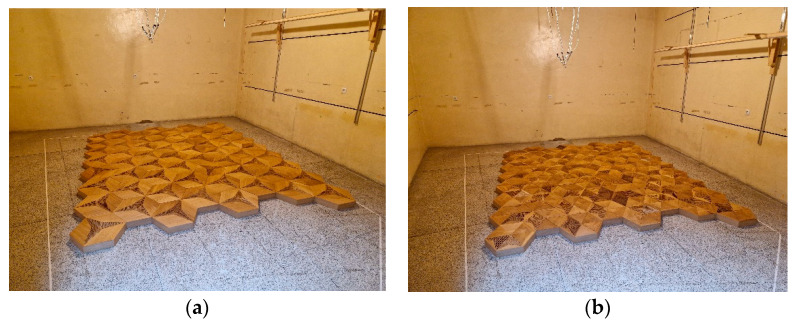
Photographs of the measured sequences in a reverberation chamber: (**a**) sequence with 100% perforated panels (CM-D); (**b**) sequence with 33% perforated panels (P-A) and 66% non-perforated panels.

**Figure 11:**
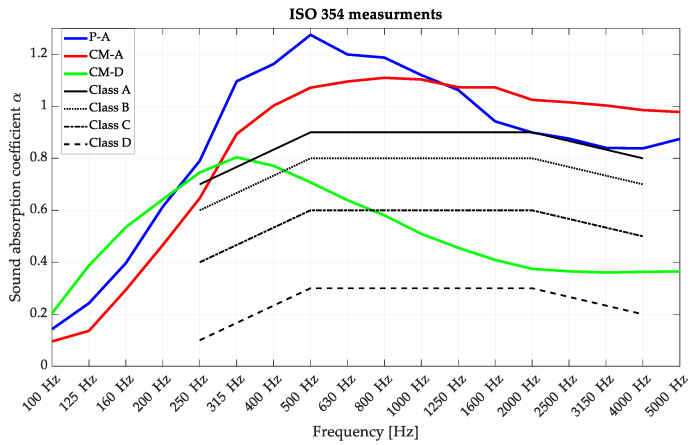
Reverberation chamber measurements of CM-A, CM-D, and P-A perforated acoustic wooden panels produced using 3rd-degree-of-freedom curves (100% of the surface was made of perforated wooden overlays).

The panels were constructed with a 50 mm layer of mineral wool serving as a porous backing with low flow resistivity. Photographs of the measured panels in the reverberation chamber at the AGH University of Science and Technology are shown in Figure 10. The hardware setup and the chamber configuration reflected the ISO 354 requirements: the reverberation chamber volume was 180.4 m^3^, the total surface was 193.6 m^2^, the air’s relative humidity was in the range of 32.3–36.4%, and the temperature was in the range of 22.2–23.1 °C throughout the measurement procedure. The sample size was 10.4 m^2^. The measurements of ISO 354 sound absorption for the overlays described in Table 1 are shown in Figure 11. They were compared with the sound absorption class borders following the ISO 11654 standard [40]. The sound absorption class is the parameter used as a single value rating for absorbing panels and is widely used by architects and interior designers; therefore, it is crucial to derive these parameters during acoustic panel certification. Detailed explanations of the sound absorption coefficients and their measurement uncertainty, single-value parameters, and acoustic classes are described in Appendix A.

The research in this stage validated the findings obtained from the numerical simulations. The P-A and CM-A overlays demonstrated Class A sound absorption characteristics calculated following ISO 11654 [40], whereas the CM-D overlay exhibited Class D sound absorption. It is crucial to emphasize that to meet the requirements for a particular class, the graph depicting the third-octave bands should predominantly exceed the threshold defined by the boundary of that class. The construction of the third-degree-of-freedom front panels allowed the perforated panels to obey the main limitation: low sound absorption in the high-frequency range. Due to this reason, these results confirm the effectiveness of the selected overlays in achieving the desired sound absorption performance, and they provide valuable insights for the design and implementation of acoustic panels in various applications. Both the P-A and CM-A panels achieved values greater than 1.0 in the mid- and high-frequency ranges. This is a well-known issue that occurs in the ISO 354 measurement procedure, which has several causes [41,42,43], such as the finite volume of the reverberation chamber, the so-called edge effect that happens on the borders and sides of the measured samples (additional absorption), the procedure for the sample surface calculations, and the incompletely diffused sound field inside the reverberation chamber. These effects cannot be obeyed, while the ISO 354 standard is required for any acoustic panel certification, and its procedure needs to be precisely followed. The most significant difference was observed between the simulated, anticipated absorption of the CM-D panel and that measured in the reverberation chamber. The source of this dispersion may have been the lack of inclusion of plate absorber behavior in the modeling process, which was visible during the measurement. The CM-D panels were constructed with single overlays such as those visible in Figure 1, so their resonant frequency may be visible in the measured frequency range of 100–200 Hz. This leads to the further conclusion that for more advanced modeling of perforated panels with a lower perforation ratio, the plate absorber mechanism needs to be considered for precise simulations.

### 4.2. Partial Application of Overlays

**Figure 12:**
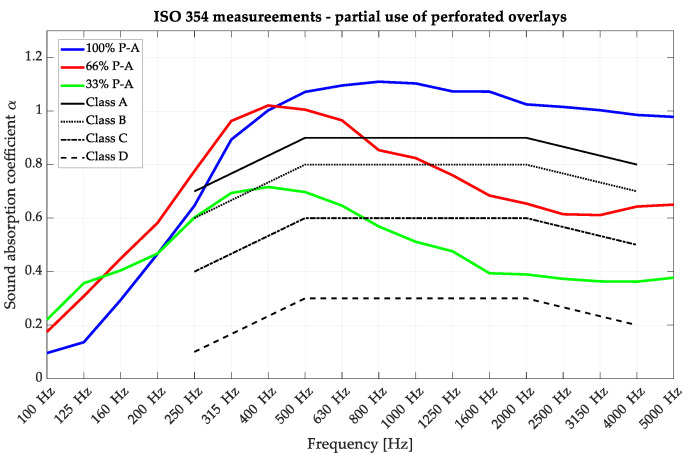
Reverberation chamber measurements of the P-A perforated acoustic wooden panels produced using 3rd-degree-of-freedom curves: variable acoustic setups with perforated overlays replaced with solid overlays.

The impact of combining solid overlays with perforated overlays was also examined to assess how these modifications would affect the measured sound absorption coefficient. The obtained results are presented in Figure 12. As observed, reducing the number of perforated overlays and replacing them with solid overlays decreased the sound absorption coefficient, shifting the sound absorption class.

The sound absorption class can be shifted between A, C, and D. The features of the replacement with the solid overlay also cover the conversion to a more resonant absorption curve, opening new possibilities for the panels’ use. Understanding the influence of the combination of non-perforated and perforated overlays provides valuable insights into the design and optimization of acoustic panels. By carefully selecting and incorporating the appropriate overlays, achieving the desired sound absorption characteristics for specific applications is possible.

## 5. Summary

This article discusses the utilization of acoustic panels with third-degree curves for shaping room acoustics and achieving the desired acoustic conditions. The comprehensive design, simulation, and measurement processes offer a guide for recreation and a proper acoustic material development process. The research process described in this article began with an overview of the available solutions and background research on the technical aspects of acoustic panels. Impedance tube tests were then conducted to measure the sound absorption coefficients of the panels, providing valuable insights for improvement. Finite element method simulations were employed to optimize the panels’ perforation rate and design parameters. The final stage involved measuring the acoustic performance of the manufactured panels in a reverberation chamber and validating the expected specifications. The contribution of this research is the development of 3D wooden perforated panels with Class A sound absorption and an overall panel depth of 90 mm (50 mm of porous backing included), significantly improving the current base of acoustic materials. The rest of the panel properties, such as the FSC wood certification and the ecological and recycling aspects, allow these materials to be used in modern architecture.

Across the presented research, we present the possible use of FEM modeling to achieve successful prototyping. It was proven that it is possible to properly simulate the behavior of acoustic materials in an FEM environment by applying the Jeong–Thomasson correction and then confirming this with reverberation chamber measurements with a preliminary prediction of the further acoustic panel performance. This method is the most suitable for acoustic material development as it is based on a numerical simulation concerning the final parameter measured. The research findings indicate that panels produced using third-degree curves with variable perforations exhibited slightly better acoustic properties than flat overlays across the measured frequency range. The 3D front panel type offered increased sound absorption in the high-frequency range, resulting in properties uncommon thus far in the state of the art of acoustic perforated materials, especially solid wood.

## Figures and Tables

**Table 1:** Calculated panel parameters showing the best efficiency.

Element (Perforation Pattern)	Number of Holes	Hole Diameter (mm)	Total Element Surface (mm^2^)	Perforation Surface (mm^2^)	Perforation Rate (%)
P-A	262	6	27,063	7408	27.37
CM-A	496	6	54,126	14,024	25.91
CM-D	224	5	54,126	7146	13.20

## Data Availability

Data available on request directly from Authors.

## Appendix A. Detailed Results and Measurement Uncertainty in Reverberation Chamber Measurements

In this section, the detailed results of the reverberation chamber measurements are presented. As the repeatability and the measurement uncertainty of the ISO 354 standard are still under study [37], detailed explanations are required. The sound absorption coefficient of acoustic panels with surface *S* is calculated as follows (A1):

(A1) $$a=55.3\frac{V}{s}\left(\frac{1}{c_{2}T_{2}}-\frac{1}{c_{1}T_{1}}\right)-4\frac{V}{s}\left(m_{2}-m_{1}\right)$$

where *c*_1_ and *c*_2_ are the sound speed values, *V* is the volume of the reverberation chamber, and *m*_1_ and *m*_2_ are the so-called power attenuation coefficients calculated as described in the ISO 9613-1 standard [44]. Very often, the *m*_1_ and *m*_2_ values can be neglected until the reverberation chamber’s atmospheric conditions are maintained. The T_1_ and *T*_2_ values are the reverberation times measured (*T*_2_—with sample; *T*_1_—empty chamber). Following the required measurement conditions, the measurement procedure generates multiple values for the reverberation time, which should be averaged. In the current research, six GRAS46AQ microphones available from AGH University of Science and Technology, Cracow, and two different omnidirectional sound source positions were used, which generated 12 values for the reverberation time for each test condition. The reverberation time’s standard deviation can be calculated as follows (A2):

(A2) $$\delta =\sqrt{\sum_{i=1}^{N}\frac{{\left(T_{i}-\bar{T}\right)}^{2}}{N\left(N-1\right)}},$$

where *N* is the number of measurements, and the spatial average of the reverberation time is (A3):

(A3) $$\bar{T}=\frac{1}{N}\sum_{i=1}^{N}T_{i},$$

The final sound absorption coefficient measurement uncertainty is then calculated with Equation (A4):

(A4) $$\delta_{a_{s}}=\frac{55.3V}{cS}\sqrt{{\left(\frac{\delta_{2}}{T_{2}^{2}}\right)}^{2}+{\left(\frac{\delta_{1}}{T_{1}^{2}}\right)}^{2}}$$

where *T*_1_ and $\delta_{1}$ are the parameters of the empty reverberation chamber, and *T*_2_ and $\delta_{2}$ are the parameters of the chamber with the test specimen. In Table A1, Table A2, Table A3, Table A4 and Table A5, the detailed sound absorption coefficient values and their uncertainty calculations are presented. The minimum and maximum values are taken from the complete set of sound absorption coefficients calculated from the reverberation time measured by a single microphone, and the uncertainty value (*U*) is defined as the standard deviation calculated using Equation (A4) multiplied by 2. The Alfa % value is the measure of the U value divided by the average sound absorption coefficient. All of the obtained values are within the allowed limits defined by the reference standards.

**Table A1:** Sound absorption coefficient calculations and their uncertainty for the CM-A panel.

CM-A—*α_w_* = 0.95 (Acoustic Class A)					
Frequency (Hz)	Alfa (-)	Min (-)	Max (-)	U	% Alfa (%)
100	0.14	0.11	0.17	0.03	22%
125	0.27	0.21	0.34	0.06	23%
160	0.45	0.41	0.49	0.04	9%
200	0.63	0.56	0.69	0.06	10%
250	0.87	0.82	0.92	0.05	6%
315	1.01	0.96	1.06	0.05	5%
400	1.08	1.02	1.13	0.05	5%
500	1.19	1.16	1.23	0.04	3%
630	1.13	1.09	1.17	0.04	4%
800	1.06	1.04	1.09	0.03	3%
1000	1.02	0.98	1.06	0.04	4%
1250	0.94	0.91	0.97	0.03	3%
1600	0.89	0.86	0.93	0.03	4%
2000	0.87	0.84	0.89	0.03	3%
2500	0.86	0.83	0.88	0.03	3%
3150	0.86	0.82	0.90	0.04	4%
4000	0.83	0.79	0.87	0.04	4%
5000	0.93	0.91	0.95	0.02	3%

**Table A2:** Sound absorption coefficient calculations and their uncertainty for the P-A panel.

P-A—*α_w_* = 0.95 (Acoustic Class A)					
Frequency (Hz)	Alfa s (-)	Min (-)	Max (-)	U	% Alfa (%)
100	0.12	0.09	0.15	0.03	24%
125	0.19	0.16	0.23	0.04	19%
160	0.35	0.31	0.39	0.04	11%
200	0.54	0.49	0.59	0.05	9%
250	0.74	0.69	0.78	0.05	6%
315	0.99	0.93	1.05	0.06	6%
400	1.08	1.04	1.12	0.04	4%
500	1.11	1.06	1.17	0.05	5%
630	1.11	1.05	1.17	0.06	5%
800	1.05	1.02	1.08	0.03	3%
1000	1.02	0.99	1.06	0.04	4%
1250	0.97	0.95	1.00	0.02	2%
1600	0.95	0.92	0.98	0.03	3%
2000	0.90	0.87	0.93	0.03	3%
2500	0.87	0.83	0.92	0.04	5%
3150	0.88	0.85	0.92	0.04	4%
4000	0.85	0.82	0.88	0.03	3%
5000	0.87	0.84	0.91	0.03	4%

**Table A3:** Sound absorption coefficient calculations and their uncertainty for the P-A 66% panel.

P-A 66%—*α_w_* = 0.75 (Acoustic Class C)					
Frequency (Hz)	Alfa s (-)	Min (-)	Max (-)	U	% Alfa (%)
100	0.17	0.14	0.21	0.03	19%
125	0.31	0.25	0.37	0.06	18%
160	0.45	0.40	0.50	0.05	11%
200	0.58	0.54	0.63	0.05	8%
250	0.78	0.72	0.83	0.05	7%
315	0.96	0.90	1.02	0.06	6%
400	1.02	0.97	1.07	0.05	5%
500	1.01	0.98	1.03	0.03	3%
630	0.97	0.92	1.01	0.05	5%
800	0.85	0.82	0.89	0.03	4%
1000	0.82	0.79	0.86	0.03	4%
1250	0.76	0.74	0.78	0.02	3%
1600	0.68	0.66	0.71	0.03	4%
2000	0.65	0.63	0.67	0.02	3%
2500	0.61	0.59	0.64	0.02	4%
3150	0.61	0.59	0.63	0.02	4%
4000	0.64	0.62	0.67	0.02	4%
5000	0.65	0.63	0.67	0.02	3%

**Table A4:** Sound absorption coefficient calculations and their uncertainty for the P-A 33% panel.

P-A 33%—*α_w_* = 0.45 (Acoustic Class D)					
Frequency (Hz)	Alfa s (-)	Min (-)	Max (-)	U	% Alfa (%)
100	0.22	0.18	0.26	0.04	16%
125	0.36	0.30	0.41	0.05	15%
160	0.40	0.37	0.44	0.04	9%
200	0.47	0.42	0.51	0.04	9%
250	0.60	0.57	0.64	0.04	6%
315	0.69	0.66	0.72	0.03	4%
400	0.72	0.67	0.76	0.05	6%
500	0.70	0.67	0.73	0.03	4%
630	0.65	0.63	0.66	0.02	3%
800	0.57	0.55	0.59	0.02	4%
1000	0.51	0.49	0.53	0.02	4%
1250	0.48	0.46	0.49	0.01	3%
1600	0.39	0.38	0.41	0.02	4%
2000	0.39	0.37	0.41	0.02	4%
2500	0.37	0.35	0.40	0.02	6%
3150	0.36	0.34	0.39	0.02	6%
4000	0.36	0.34	0.39	0.02	6%
5000	0.38	0.36	0.39	0.01	4%

**Table A5:** Sound absorption coefficient calculations and their uncertainty for the CM-D panel.

CM-D—*α_w_* = 0.45 (Acoustic Class D)					
Frequency (Hz)	Alfa s (-)	Min (-)	Max (-)	U	% Alfa (%)
100	0.20	0.16	0.22	0.05	25%
125	0.39	0.33	0.42	0.04	10%
160	0.54	0.51	0.58	0.04	7%
200	0.64	0.6	0.67	0.04	6%
250	0.75	0.72	0.77	0.03	4%
315	0.80	0.75	0.82	0.04	5%
400	0.77	0.75	0.81	0.04	5%
500	0.71	0.68	0.73	0.03	4%
630	0.64	0.61	0.66	0.02	3%
800	0.58	0.55	0.6	0.03	5%
1000	0.51	0.49	0.53	0.02	4%
1250	0.46	0.43	0.47	0.02	4%
1600	0.41	0.4	0.43	0.01	2%
2000	0.37	0.36	0.38	0.01	3%
2500	0.37	0.35	0.39	0.02	6%
3150	0.36	0.35	0.38	0.01	3%
4000	0.36	0.34	0.37	0.02	6%
5000	0.36	0.35	0.37	0.01	4%
